# Midwifery

**Published:** 1829-05

**Authors:** 


### MIDWIFERY.

*Twin Case; Inflammation of the Womb, and of the Cellular Membrane surrounding the broad Ligaments; considerable Deposition of Pus in the Ovarian, the right Iliac, and the inferior Cava Veins.* (HOSPICE DE PERFECTIONNEMENT.)

M. GUILLERY was sent for, on the 25th of December, 1828, about half past twelve at night, to attend Adelaide C. Blondeau, ætat. twenty-seven, in her first labour. Hemorrhage from the vagina had occurred; and examination showed that the os uteri was soft, elongated, yet not dilated. At three o'clock dilatation commenced; the pains followed each other quickly, and the membranes protruded. The patient was reduced to the lowest ebb of misery; the great fatigue she had undergone during pregnancy, the privations of all kinds she had suffered through poverty, and the anxieties to which she had been a prey, all concurred to make a tedious labour, and unfavorable consequences, matters to be feared; therefore it was proposed to remove her to the Hospice de Perfectionnement, where she arrived about eleven o'clock.

She was immediately put upon the labour bed. The membranes became more and more protuberant, and passed beyond the labia externa, notwithstanding the absence of pains. At six in the evening, the woman, tired of remaining in the same position, attempted to get up; but the membranes quickly gave way, and the waters were discharged. She was replaced on the bed; pains came on, and at nine P.M. a vertex presentation was evident; a strong contraction completely expelled a male child, of considerable size; the funis was then tied and divided as usual.

The abdomen of the woman remaining large, another examination was made, which ascertained the head of a second fœtus: there was a little bloody discharge from the vagina. The uterus was again inactive. The ergot of rye was on the point of being given: the woman was, however, first made to walk about, and towards eleven o'clock the pains returned, became stronger and stronger; about midnight they followed each other rapidly, and a second infant was born, (a female.) To remove the afterbirth, traction was made on both cords at once. The placentas were separate; that of the last-born infant came away first, the other followed soon after. But little hemorrhage succeeded.

26th.—The patient is doing well. The uterus is large; the lochial discharge abundant. Broth for diet.

27th.—In the same state.

28th.—The milk troublesome; the breasts very large. Fever diet.

30th.—The fever remains. Breasts very large and very hard; the uterus a little contracted; abdomen large, yet not painful; parietes relaxed.—Let slight compression be made with a napkin for a bandage.

31st.—Lochia less.—Let poultices be applied to the abdomen and upper and inner part of the thighs, and let them be renewed twice a day. Broth and diluents.

January 1st and 2d, 1829.—Same state.—Same prescriptions, with the addition of emollient clysters.

3d.—Patient quiet. In the evening the lochia ceased.—M. Guerseul had fifteen leeches applied to the labia externa; loss of blood estimated at twelve ounces in half an hour.

5th.—In the morning visit, the patient complained of pain in the iliac fossa, and said that she had suffered the like long before her accouchement.—Twelve leeches to each part in pain. The flow of blood was so great, that it was obliged to be stopped in the evening.

6th.—A little better.—Let her go into the warm bath for half an hour. Continue the cataplasms.—Abdomen tense; no stool. Haust. oleos. vespere.

7th.—Several evacuations from the draught. Repeat the clysters.

8th, vespere.—Pulse very frequent, 120; she has cough, which returns in fits, chiefly when she is sitting up.—Let her be bled to eight ounces.

9th.—Pulse less frequent; cough gone; countenance anxious, eyes haggard. Desired greatly to sit up, and remained half an hour in an easy chair. She has had a purging.

10th.—Delirious during the night; evacuations passed under her. She endeavours to get up, and at times is extremely impatient and ungovernable. The left upper eyelid and the nose are a little puffed, foreshowing that these parts will become attacked with erysipelas: this is thought a good sign, and it is not proposed to counteract its progress.

11th, in the morning.—Erysipelas develops itself slowly. The patient has rested little during the night; the fever is constant, and very high. She has lemonade for drink. She remained an hour in an easy chair, and will not lie down of her own accord. By the evening the erysipelas had increased; it occupied the whôle face; but it is singularly contrasted with ordinary erysipelas, for the swelling is not accompanied with any redness.—Broth. Let poultices be applied to the back and to the soles of the feet; let them be slightly irritating.

12th.—She has passed a very restless night. She has been delirious, and says she has a thick veil over her eyes, which prevents her seeing the light. The progress of the erysipelas is not favorable.—Poultices to the calves of the legs; same drink.

13th.—Let blisters be applied to the inner part of each leg. The night has been more calm, although there has been delirium. —Let a free evacuation be obtained by clysters.

14th.—The night has been spent pretty tranquilly. She has passed several stools under her. Blisters risen well. Erysipelas confined to the lips and upper eyelids, of which the right can scarcely be raised. The leechbites in the iliac regions are deeply ulcerated; the skin and cellular tissue are destroyed, and the aponeurosis may be seen. The circumference of the ulcerations is neither inflamed nor painful, which is an evil omen: they are dressed with lint and cerate.

15th and 16th.—Same condition. The thighs and legs are œdematous.

17th.—Pulse bad. Let blisters be applied to the inside of each thigh.

In the evening the extremities grew cold; and the patient died at five A.M. on the 18th.

*Examination, thirty-six hours after death.*—External appearances: General discoloration; ulcerations on the front part of the pelvis.

Internal appearances: Brain not examined.

Respiratory system: Larynx, trachea, and bronchi healthy; lungs crepitating, no tubercles; old adhesions of the pleura pulmonalis on both sides to the pleura costalis.

Circulating system: Heart and large vessels healthy; but the right iliac vein entirely filled with a solid white pus, which rose in the cava almost as high as the kidney: the internal surface of these vessels covered with false membranes. The ovarian veins equally filled with pus.

Digestive organs: Pharynx and œsophagus present no alterations; stomach pale and injected in patches, its mucous membrane not softened. The other organs presented nothing worthy of note.

Organs of generation: Uterus firmly contracted, lodged in the lower pelvis; the os almost entirely closed; the substance of this organ a little softened, as well as its internal membrane; the vessels which ramify within its walls contain no pus. The mucous membrane of the vagina reddish; both labia swelled.

Peritoneum: No adhesion observed between this membrane and the intestines; it does not appear to have suffered from inflammation.*

